# Suppression Technique of HeLa Cell Proliferation Using Ultrasonic Power Amplifiers Integrated with a Series-Diode Linearizer

**DOI:** 10.3390/s18124248

**Published:** 2018-12-03

**Authors:** Se-woon Choe, Hojong Choi

**Affiliations:** Department of Medical IT Convergence Engineering, Kumoh National Institute of Technology, Gumi-Daero 350-27, Gumi 39253, Korea; sewoon@kumoh.ac.kr

**Keywords:** ultrasonic power amplifiers, series-diode linearizer, proliferation, ultrasonic transducers

## Abstract

A series-diode linearizer scheme is developed, which can possibly generate higher voltage signals. To verify our proposed concept, ultrasonic power amplifiers with and without the linearizer were tested for HeLa cells proliferation in vitro. In general, ultrasonic stimulus initiates the process of cavitation which can cause cell lysis and disruption of cell attachment. The cavitation can also induce formation of free radicals so that a rigid membrane of malignant cancer cells have increased sensitivity to ultrasonic stimulus. The cell density of the control group increased up to almost 100% on Day 3. However, cell densities of the experimental group when using an isolated ultrasonic power amplifier, and ultrasonic power amplifiers integrated with the linearizer at 1 V and 5 V DC (direct current) bias could be suppressed more than that when using an ultrasonic power amplifier (90.7 ± 1.2%, 75.8 ± 3.5%, and 68.1 ± 1.1%, respectively). Additionally, the proliferation suppressing ratios of each experimental group confirmed that the cell density decrements of the experimental groups exhibited statistical significance compared to the control group (ultrasonic power amplifier = 8.87%, ultrasonic power amplifier with 1 V biased linearizer = 23.87%, and ultrasonic power amplifier with 5 V biased linearizer = 31.56%).

## 1. Introduction

Ultrasound systems are widely used in non-destructive testing, medical imaging, and therapeutic ultrasound applications [1,2,3]. Recently, the therapeutic ultrasound has been developed based on the advanced ultrasonic transducer and semiconductor technology [4]. The therapeutic ultrasound can be divided into two major categories: low-level (<3 W/cm^2^) and high-level (≥3 W/cm^2^) therapeutic ultrasounds [5]. The high-level therapeutic ultrasound, which is also called high-intensity focused ultrasound (HIFU), is used to treat solid cancer tissues (>1 cm^2^), considering all safety regulations [6]. In this type of therapeutic ultrasound, acoustic waves that are produced due to mechanical effects and acoustic cavitation are focused into the target, causing the thermal evaporation of the target, and thus destroying the tumors [7]. Compared to the high-level therapeutic ultrasound, the low-level therapeutic ultrasound causes mechanical effects that can be easily applied to produce functional changes in tissues or cells without much consideration of safety regulations [5]. Briefly, ultrasonic stimulus by the low-level intensity initiated the process of cavitation that causes cell damage [5]. The process of cavitation can directly cause cell lysis or the disruption of cell attachment for cell proliferation. Most cancer cells have a more rigid membrane compared to normal cells that is more sensitive to ultrasonic stimulus. In addition, the cavitation often forms free-radicals able to disrupt cellular membranes. Therefore, it can be used to suppress cancer cell proliferation, which is one of the functional changes, and thus improve the anticancer chemicals [8]. In particular, the low-level therapeutic ultrasound typically works in nonlinear acoustic ranges, which cannot effectively predict various behavior models in tissue or cell levels [9]. Additionally, a certain power or voltage level triggered ultrasonic transducers in the low-level therapeutic ultrasound have relatively limited ranges as compared to the ones in the high-level therapeutic ultrasound. This restricts the application of effective solutions even though the low-level ultrasound can be easily regulated [5]. Therefore, the design of an ultrasound system for low-level therapeutic ultrasounds to improve the therapeutic effects is an important issue to be considered.

Ultrasound systems are typically composed of ultrasonic transmitters, transducers, and receivers [10,11]. The ultrasonic power amplifiers are the last stage ultrasonic transmitter components and are one of the most critical electronic components, which directly transfer the electrical signals or power the ultrasonic transducers [2,12]. Parameters such as harmonic distortions, voltage gains, and bandwidths affect the performance of ultrasonic transducers in ultrasonic transmitters [4,10]. Recently, linearizer schemes have been introduced in the ultrasound systems to improve the image quality of the received echo signals by minimizing the harmonic distortions of triggering transmit signals of the ultrasonic power amplifiers [13,14]. The previous approach reduces the harmonic distortions of the echo signals for ultrasonic imaging applications. However, our proposed approach could rather increase the transmitted power gains for therapeutic applications. Therefore, we propose a series-diode linearizer scheme developed to serve therapeutic purposes so that it can help an ultrasonic power amplifier effectively transfer the signals at a certain frequency. The electrical impedances of the series-diode linearizer, controlled by external DC voltage, can change the electrical impedances of an ultrasonic power amplifier, thus matching the electrical impedances of an ultrasonic transducer with more freedom. Consequently, ultrasonic power amplifiers can produce high-voltage signals to drive higher power to the ultrasonic transducers, thus effectively transferring the higher ultrasonic waves into the target.

**Figure 1:**
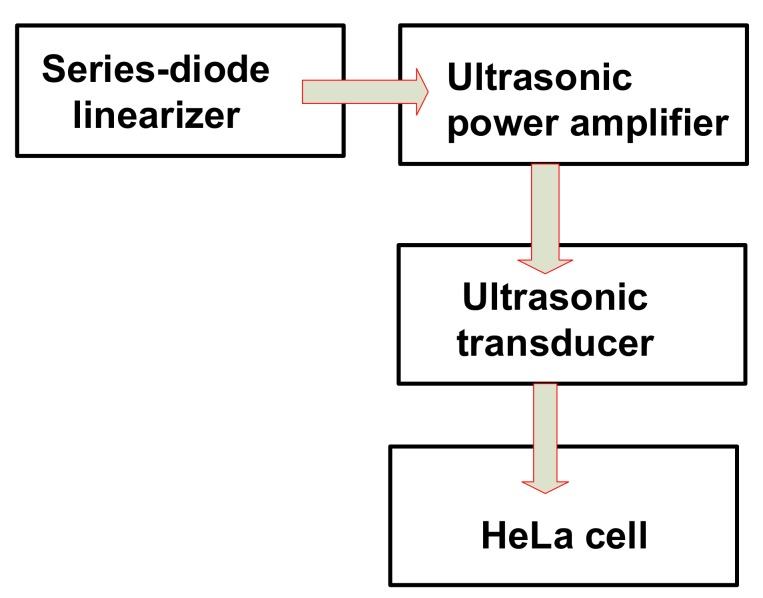
Strategic representation of an ultrasonic power amplifier integrated with a series-diode linearizer.

An electrical impedance matching circuit, which is placed between ultrasonic power amplifiers and ultrasonic transducers, can be used to improve the performance parameters, such as voltage gains and bandwidths, of ultrasonic transducers [4]. An impedance transformer has been used to obtain electrical matching to improve the voltage gains or bandwidths of an air-coupled type ultrasonic transducer that has a high value of the electrical impedances [15]. A low-pass filter has also been used to obtain electrical matching with an ultrasonic transducer to improve the bandwidths [16]. These techniques involve the use of electrical components that are relatively high-voltage tolerant and might affect the center frequency of ultrasonic transducers owing to the high values of the inductances. These techniques also might affect the ring down of the transmit ultrasonic pulses for ultrasonic transducers [17]. Since the proposed series-diode linearizer is placed in the front of the ultrasonic power amplifier, as shown in Figure 1, it does not require high-voltage tolerant components and does not critically affect the center frequency of the ultrasonic transducers. Additionally, an external DC voltage in the proposed series-diode linearizer can change the electrical impedances of the ultrasonic power amplifiers with more freedom, thus being matched with the electrical impedances of the ultrasonic transducer. Therefore, higher transmitted voltages from the ultrasonic transducers could be transferred into the desired target.

## 2. Materials and Methods

**Figure 2:**
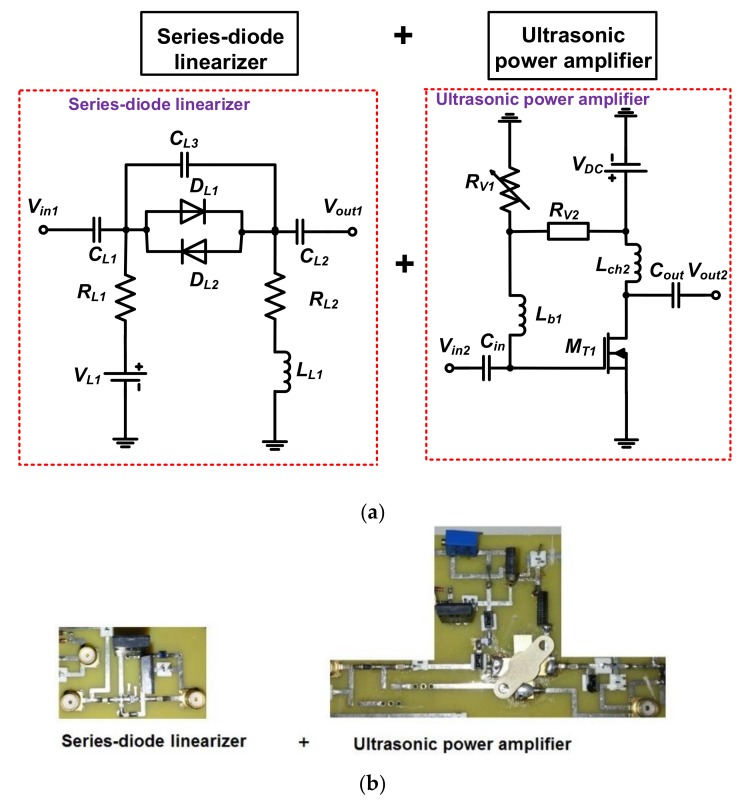
(**a**) Schematic diagrams; (**b**) implemented printed circuit board of the ultrasonic power amplifier and series-diode linearizer.

Figure 2a shows the schematic diagrams of the DC voltage driven series-diode linearizer and ultrasonic power amplifier. The ultrasonic power amplifier is a typical power amplifier [18]. In the series-diode linearizer circuit, single cross-coupled diodes (*D*_1_ and *D*_2_) were used to transmit bipolar, multicycle pulse waveforms into the ultrasonic power amplifier. The cross-coupled diode impedances can be changed depending on the applied DC bias voltages. Three capacitors (*C_L_*_1_, *C_L_*_2_, and *C_L_*_3_) and two resistors (*R_L_*_1_ and *R_L_*_2_) were used to minimize the pulse fluctuation at the input port (*V_in_*_1_). An additional inductor (*L_L_*_1_) was used to compensate the capacitances (*C_L_*_1_, *C_L_*_2_, and *C_L_*_3_).

In the ultrasonic power amplifier, two input and output coupling capacitors (*C_in_* and *C_out_*) were used because high-voltage DC can generate long discharged pulses when receiving the echo signals from the ultrasonic transducers. The choke inductor (*L_ch_*_2_) was used to reduce DC voltage variances. The variable and fixed resistors (*R_v_*_1_ and *R_v_*_2_) were used to provide gate bias voltage to the primary transistor (*M_T_*_1_) from the DC power supply (*V_DC_*). Figure 2b shows the implemented series-diode linearizer and ultrasonic power amplifier, which were connected through a short SMA coaxial cable of 20 cm in length to minimize the signal distortions between them. A heat sink is attached at the bottom of the transistor in the printed circuit board to dissipate heat when operating the high-voltage pulses for therapeutic applications.

**Figure 3:**
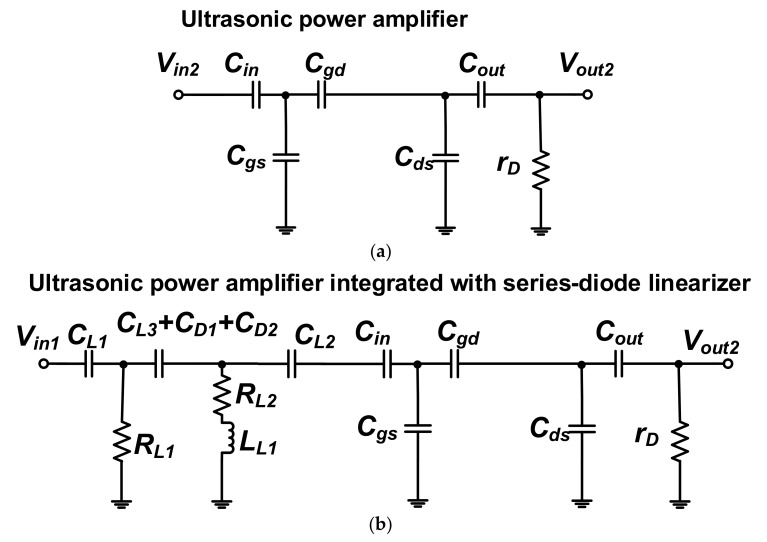
Equivalent circuit models of (**a**) the isolated ultrasonic power amplifier; (**b**) the ultrasonic power amplifier integrated with series-diode linearizer.

Figure 3 shows the equivalent circuit models of the isolated ultrasonic power amplifier and the ultrasonic power amplifier integrated with the series-diode linearizer to show electrical impedance variances after consolidating the series-diode linearizer circuit.

The electrical impedances of the ultrasonic power amplifier (*Z_tran_*) and the ultrasonic power amplifier integrated with the series-diode linearizer (*Z_tran+lin_*) were expressed in Equations (1) and (2).

(1) $$Z_{tran}=\frac{1}{\frac{1}{r_{D}}+j2\pi f_{c}\left(C_{in}+C_{gs}+C_{gd}+C_{ds}+C_{out}\right)}$$

(2) $$Z_{tran+lin}=\frac{1}{\frac{1}{r_{D}}+j2\pi f_{c}\left(C_{in}+C_{gs}+C_{gd}+C_{ds}+C_{out}\right)+\frac{R_{L1}}{1+j2\pi f_{c}(C_{1}R_{L1})}+\frac{1}{1+j2\pi f_{C}R_{L2}-4\pi^{2}f_{c}{}^{2}C_{L2}L_{L1}}}$$

where *r_D_*, *C_gs_*, *C_gd_*, and *C_ds_* are parasitic drain resistance, gate-source, gate-drain, and drain-source capacitances, respectively, of the primary transistor (*M_T_*_1_), *C*_1_ is the combined capacitance of the diode parasitic capacitances (C_D1_ and C_D2_) with capacitor (*C_L_*_3_), and *f_c_* is the operating frequency of the ultrasonic power amplifier.

As already mentioned, an inductor (*L_L_*_1_) and two resistors (*R_L_*_1_ and *R_L_*_2_) were used to compensate the capacitances of the cross-coupled diodes in the series-diode linearizer, and parasitic resistances and capacitances of the ultrasonic power amplifier at a certain frequency. Therefore, electrical impedances of the ultrasonic power amplifier integrated with the series-diode linearizer can be changed further as compared to those of the isolated ultrasonic power amplifier. According to Equation (2), the electrical impedances could be adjustable because the electrical impedances of the ultrasonic power amplifier integrated with series-diode linearizer (Z_tran+lin_) could be varied depending on the *f_c_* and *C*_1_ if the fixed resistors (*R_L_*_1_ and *R_L_*_2_), capacitors (*C_L_*_1_, *C_L_*_2_, and *C_L_*_3_), and inductor (*L_L_*_1_) in the series-diode linearizer were selected. At the fixed operating frequency (*f_c_*), the electrical impedances are dependent on the diode capacitance (*C*_1_) due to fixed resistances, capacitances, and inductance. Therefore, we can confirm that additional DC voltage (*V_L_*_1_) in the series-diode linearizer could change the electrical impedances of the ultrasonic power amplifier. Next, the measured electrical impedances are compared with the ultrasonic power amplifier with and without series-diode linearizer.

To study the therapeutic effects of the proposed ultrasonic power amplifier with and without the series-diode linearizer, the cell densities and proliferation suppressing ratios (PSRs) were measured on the human cervical cancer cell (HeLa cells, Korean Cell Line Bank) in vitro. HeLa cells were maintained in a high-glucose Dulbecco’s Modified Eagle Medium containing 10% Fetal Bovine Serum with 1% penicillin streptomycin and incubated at 37 °C in a humidified benchtop incubator with 5% CO_2_. The prepared cells were trypsinized when the growing cells reached confluence at 70% and then washed three times with phosphate-buffered saline to isolate them in the media. Next, they were resuspended to prepare an approximate concentration of $1\times 10^{6}$ cells/mL. When the cell confluence reached 40%, the ultrasonic stimulus was induced and consequently counted as Day 0. To avoid thermal damages, the ambient temperature was adjusted to 26 °C during the preparation and experimental procedures. A probe container printed using a commercial 3D printer (Cubicon 3DP-310F, Cubicon Inc., Gyeonggi-do, Korea), was designed to hold the ultrasound transducer. It stimulated the same previous spot on the prepared culture dish. The ultrasonic transducer was placed in the surface of the growth media in the cell culture dish, and its position was retained until Day 3. All prepared samples were divided into four groups: control group (no ultrasonic induction, *n* = 6), ultrasonic power amplifier group (ultrasonic induction with ultrasonic power amplifier, *n* = 6), ultrasonic power amplifier integrated with series-diode linearizer 1 V (when 1 V DC bias voltage is applied, *n* = 6), and ultrasonic power amplifier integrated with series-diode linearizer 5 V (when 5 V DC bias voltage is applied, *n* = 6). The ultrasonic stimulus was induced daily for about 30 min. To quantify cell densities and PSRs, the microscopic images of the ultrasound signal-focused area in the cell culture dish were taken instantly after ultrasound signal induction by an inverted fluorescent microscope (IX73 with DP80, Olympus Corp., Tokyo, Japan). Various image processing procedures and statistical analyses using ANOVA with Scheffe’s post-hoc test were performed using the MATLAB software (MathWorks, Natick, MA, USA) to identify the quantitative characteristics of each group [19]. The cell density was determined by dividing the pixel number of the cell grown area by the acquired microscopic pixel number. The PSR was calculated by dividing the variance of cell density of the experimental group and the control group by the cell density of the control group on Day 3. A *p* value of less than 0.05 was considered statistically significant as compared to the control group.

## 3. Results and Discussion

### 3.1. Performances of the Series-Diode Linearizer and Ultrasonic Power Amplifier

**Figure 4:**
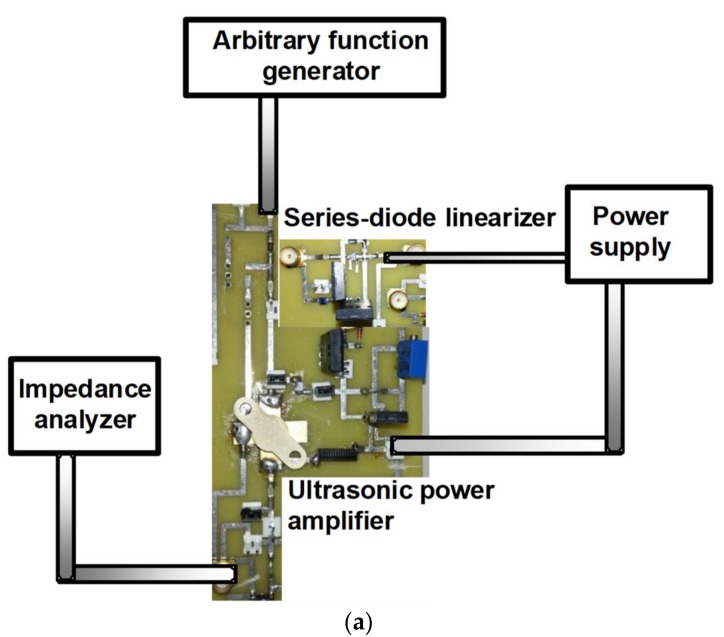

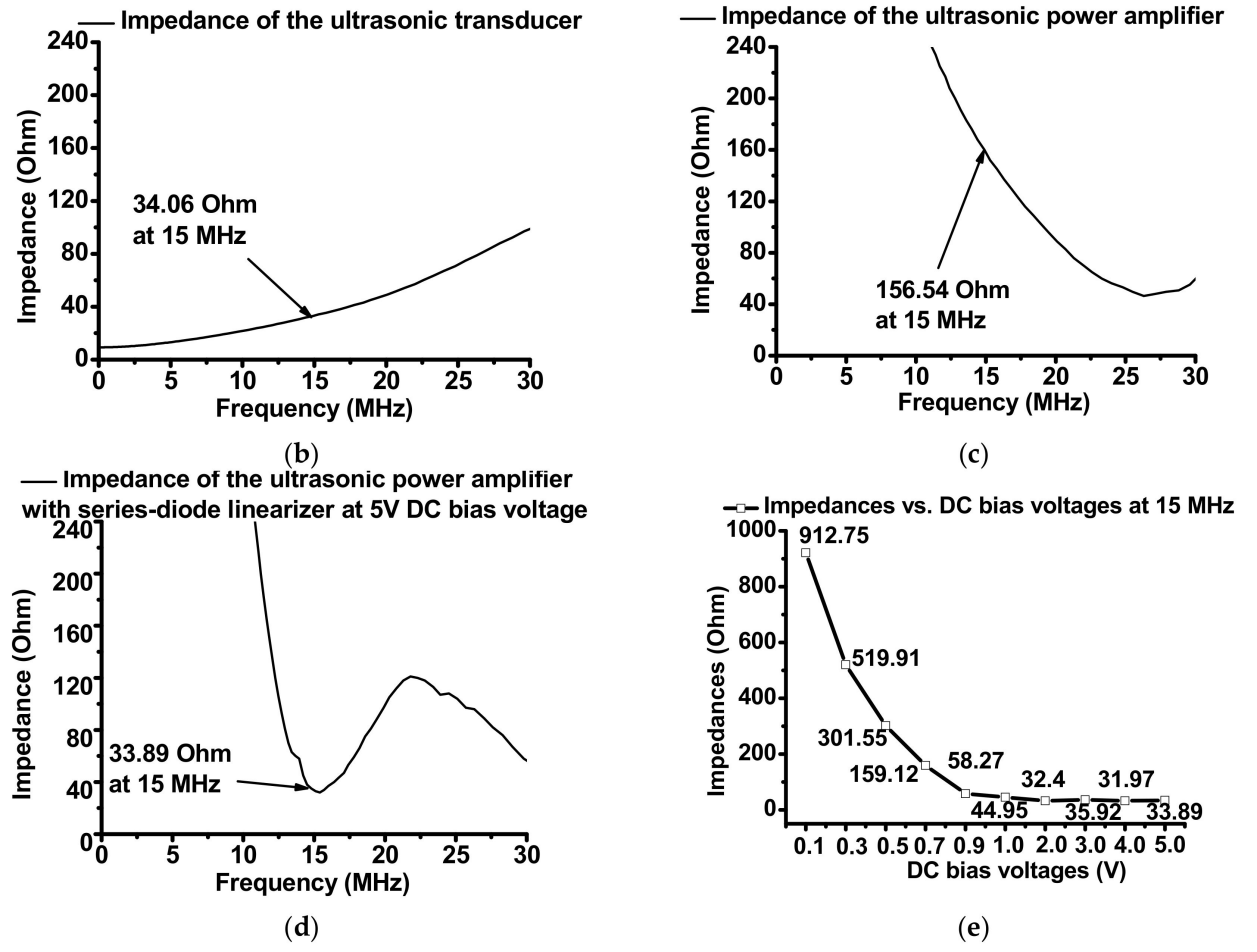
(**a**) Measurement setup of electrical impedances of the isolated ultrasonic power amplifier and the ultrasonic power amplifier integrated with series-diode linearizer; (**b**) electrical impedances of 15 MHz ultrasonic transducer; (**c**) the isolated ultrasonic power amplifier; (**d**) ultrasonic power amplifier integrated with series-diode linearizer at 5 V DC; (**e**) electrical impedances of the ultrasonic power amplifier with series-diode linearizer at 0.1 V, 0.3 V, 0.5 V, 0.7 V, 0.9 V, 1 V, 2 V, 3 V, 4 V, and 5 V DC bias voltages at 15 MHz.

Figure 4a shows the measurement setup of electrical impedances of the isolated ultrasonic power amplifier and the ultrasonic power amplifier integrated with the series-diode linearizer at different DC bias levels in the impedance analyzer. A 15 MHz and bipolar sine waveform with a variable input power level generated from an arbitrary function generator (AFG3101C, Tektronix Inc., Beaverton, OR, USA) was the inputs of the isolated ultrasonic power amplifier and the ultrasonic power amplifier integrated with series-diode-linearizer. The output port from the ultrasonic power amplifier integrated with a series-diode-linearizer was connected to the input port of the impedance analyzer. This measurement shows the impedance variances of the series-diode linearizer. In Figure 4b,c, the measured electrical impedances of ultrasonic transducer and ultrasonic power amplifier are 34.06 Ω and 156.54 Ω, respectively, at 15 MHz. However, the electrical impedance of 34.06 Ω of the ultrasonic transducer is not used in typical electronic devices that require an electrical impedance of 50 Ω. Therefore, we need to develop a series-diode linearizer that can change the electrical impedances of the ultrasonic power amplifier to match it with that of the ultrasonic transducers. In Figure 4d, the measured electrical impedances of the ultrasonic power amplifier integrated with the series-diode linearizer at 5 V DC bias voltage (33.89 Ω) are close to that of the ultrasonic transducer at 15 MHz (34.06 Ω). As shown in Figure 4e, the electrical impedances of the ultrasonic power amplifier integrated with the series-diode linearizer at 0.1 V, 0.3 V, 0.5 V, 0.7 V, 0.9 V, 1 V, 2 V, 3 V, 4 V and 5 V DC bias voltages were measured to show the variances of the electrical impedances. The obtained electrical impedances could be changed from 912.75 Ω to 31.97 Ω with respect to the applied DC bias voltages. The measured impedances of the ultrasonic power amplifier integrated with the series-diode linearizer at 1 V–5 V DC bias voltages are also close to that of the ultrasonic transducer at 15 MHz since one of the main components in the series-diode linearizer, which changes the electrical impedances, is a diode working at over 1 V DC. Therefore, we need to apply any DC bias voltage of over 1 V DC to match the electrical impedances of the ultrasonic transducers.

**Figure 5:**
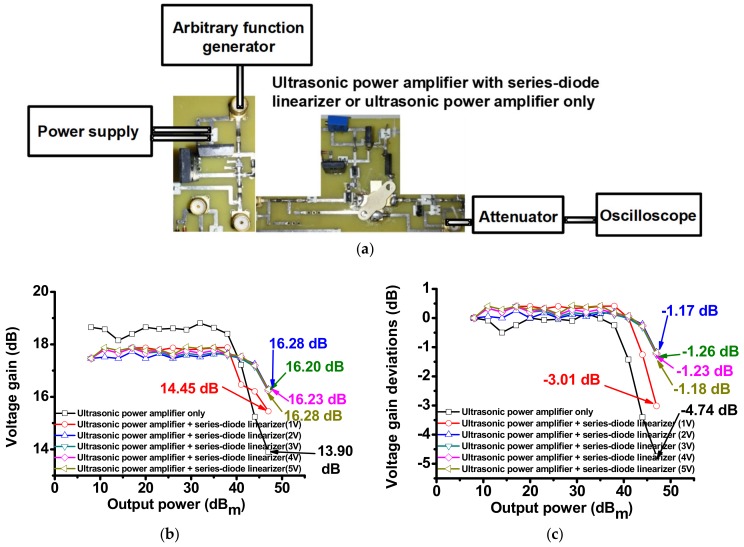
(**a**) Measurement setup; (**b**) voltage gain; (**c**) voltage gain deviation of the isolated ultrasonic power amplifier and the ultrasonic power amplifier integrated with series-diode linearizer at 1 V, 2 V, 3 V, 4 V, and 5 V DC bias voltages.

Figure 5a shows the measurement setup and measured voltage gain graphs of the isolated ultrasonic power amplifier and the ultrasonic power amplifier integrated with the series-diode linearizer. A 15 MHz and bipolar sine waveform with a variable input power level generated from an arbitrary function generator (AFG3101C) was the inputs of the isolated ultrasonic power amplifier and the ultrasonic power amplifier integrated with a series-diode-linearizer. A 50 W attenuator (BW-N40W50+, Mini-Circuits, Brooklyn, NY, USA) was inserted to measure peak-to-peak voltages of the output voltage waveforms since a 50 Ω impedance setting of the oscilloscope (MSO4024B, Tektronix Inc., Beaverton, OR, USA) has a maximum voltage of 5 V.

Figure 5b,c show the measured voltage gain and voltage gain deviations of the isolated ultrasonic power amplifier and the ultrasonic power amplifier integrated with the series-diode linearizer. 15 MHz input bipolar sine pulses generated from an arbitrary function generator, were applied to the isolated ultrasonic power amplifier and the ultrasonic power amplifier integrated with the series-diode linearizer at 1 V, 2 V, 3 V, 4 V, and 5 V DC bias voltages. The peak-to-peak output voltages were measured to calculate the voltage gain and voltage gain deviation of the isolated ultrasonic power amplifier and the ultrasonic power amplifier integrated with the series-diode linearizer. As shown in Figure 5b,c, the highest voltage gain and the voltage gain deviation of the ultrasonic power amplifier were measured as 13.90 dB and −4.74 dB, respectively. In Figure 5b,c, the highest voltage gain and voltage gain deviation of the ultrasonic power amplifier integrated with the series-diode-linearizer at 1 V DC was measured as 14.45 dB and −3.01 dB, respectively. This measurement data confirms that the series-diode linearizer with a certain DC bias voltage can boost the voltage gain at a higher output power and reduce the voltage reduction in the wide output power ranges. This can be desirable for the ultrasonic transducers that need to be triggered by high-voltage signals.

### 3.2. Performances of the Pulse-Echo Responses Using Ultrasonic Power Amplifier Integrated with Series-Diode Linearizer

**Figure 6:**
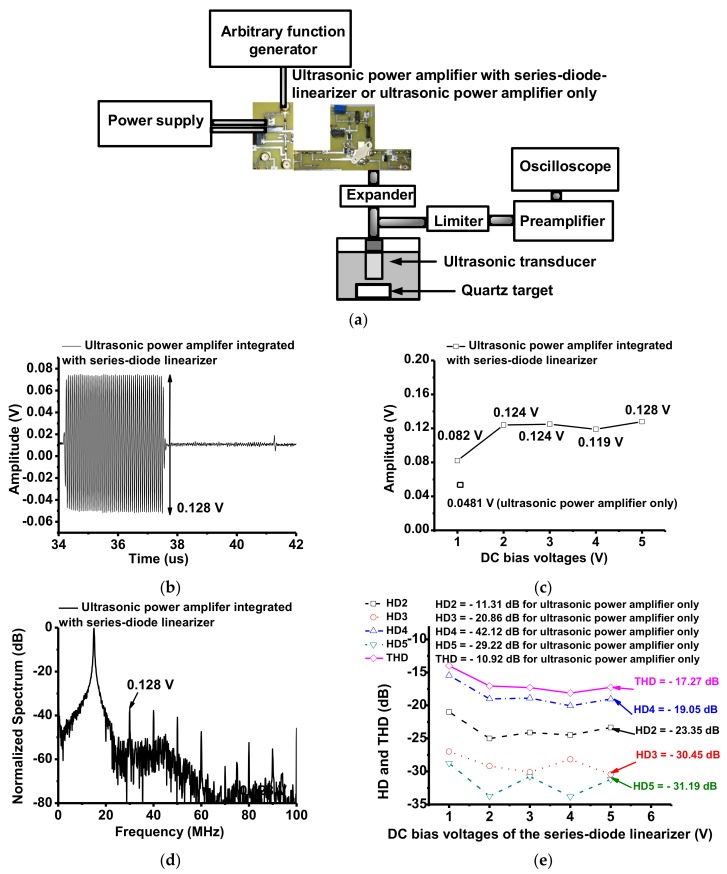
(**a**) Pulse-echo response setup; (**b**) Echo amplitude when using the ultrasonic power amplifier integrated with series-diode linearizer; (**c**) Echo amplitude comparison data when using the ultrasonic power amplifier with and without series-diode linearizer at 5 V DC bias voltage; (**d**) Spectrum data when using the ultrasonic power amplifier integrated with series-diode linearizer at 5 V DC bias voltage; (**e**) HD and THD comparison data of the echo signals of the ultrasonic transducers when using ultrasonic power amplifiers integrated with series-diode linearizer and the isolated ultrasonic power amplifier only.

Figure 6a shows the measurement setup of pulse-echo responses when using the ultrasonic power amplifiers with and without the developed series-diode linearizer to confirm that the capability of the series-diode linearizer can match that of the ultrasonic transducer while boosting the voltage gain at a higher power output. 15 MHz input bipolar sine pulses generated from an arbitrary function generator (AFG3101C) were applied to the isolated ultrasonic power amplifier and the ultrasonic power amplifier integrated with the series-diode linearizer at 1 V, 2 V, 3 V, 4 V, and 5 V DC voltages. Amplified output power passed through an expander, composed of a series of cross-coupled diodes that paired to the 15 MHz ultrasonic transducer. Received echo signals generated from the ultrasonic transducer were passed through the limiter and then, amplified by a preamplifier (AU-1114, MITEQ Inc., Hauppauge, NY, USA). The echo signals were displayed on the oscilloscope (MSO4024B). In Figure 6b, the measured echo amplitude when using the ultrasonic power amplifier integrated with the developed series-diode linearizer at 5 V DC voltage was 0.128 V_p-p_. As shown in Figure 6c, the measured echo amplitude when using the isolated ultrasonic power amplifier was 0.0481 V_p-p_ since the series-diode linearizer after 1 V DC voltages could reduce the voltage reduction in higher output power ranges as shown in Figure 5. The measured echo amplitudes when using the ultrasonic power amplifier with the developed series-diode linearizer vary from 0.082 V_p-p_ to 0.128 V_p-p_, which are very constant amplitude levels above 1 V DC bias voltage and around 2.5 times as compared to when using the isolated ultrasonic power amplifier. Figure 6d shows the measured spectrum data when using the ultrasonic power amplifier integrated with the developed series-diode linearizer.

Figure 6e shows the comparison of 2nd, 3rd, 4th, and 5th harmonic distortions and total harmonic distortion (HD2, HD3, HD4, HD5, and THD) values in the spectrum data to show distortions of the echo signals. The measured HD2, HD3, HD4, HD5, and THD values, when using the isolated ultrasonic power amplifier are −11.31 dB, −20.86 dB, −42.12 dB, −29.22 dB, and −10.92 dB, respectively. The measured HD2, HD3, HD4, HD5, and THD values when using the ultrasonic power amplifier integrated with the series-diode linearizer at 5 V DC bias voltage were reduced to −23.35 dB, −30.45 dB, −19.05 dB, −31.19 dB, and −17.27 dB, respectively. Even though the DC bias voltages of the series-diode linearizer were different, the echo amplitude and THD values have variance less than 0.005 V_p-p_ and 1.2 dB above 1 V DC bias voltage such that the performances are almost constant. Since the variance of the resistance of the diode above 1 V DC is almost constant, the electrical impedances of the series-diode linearizer can be similar. When using the ultrasonic power amplifier integrated with the series-diode linearizer, HD4 values are worse but the THD values more than 6 dB are enhanced. This data indicates that the series-diode linearizer can help the ultrasonic power amplifier match the ultrasonic transducer. It is also expected that the ultrasonic power amplifier integrated with the series-diode linearizer can produce a higher power for the target.

### 3.3. Performances of the HeLa Cells Proliferation Suppression

**Figure 7:**
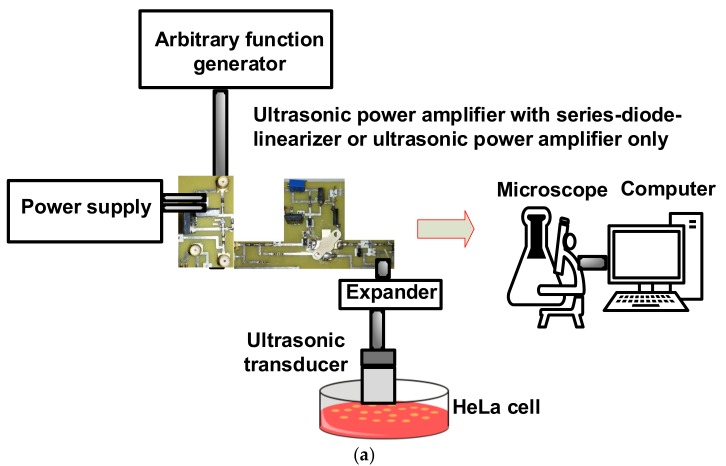

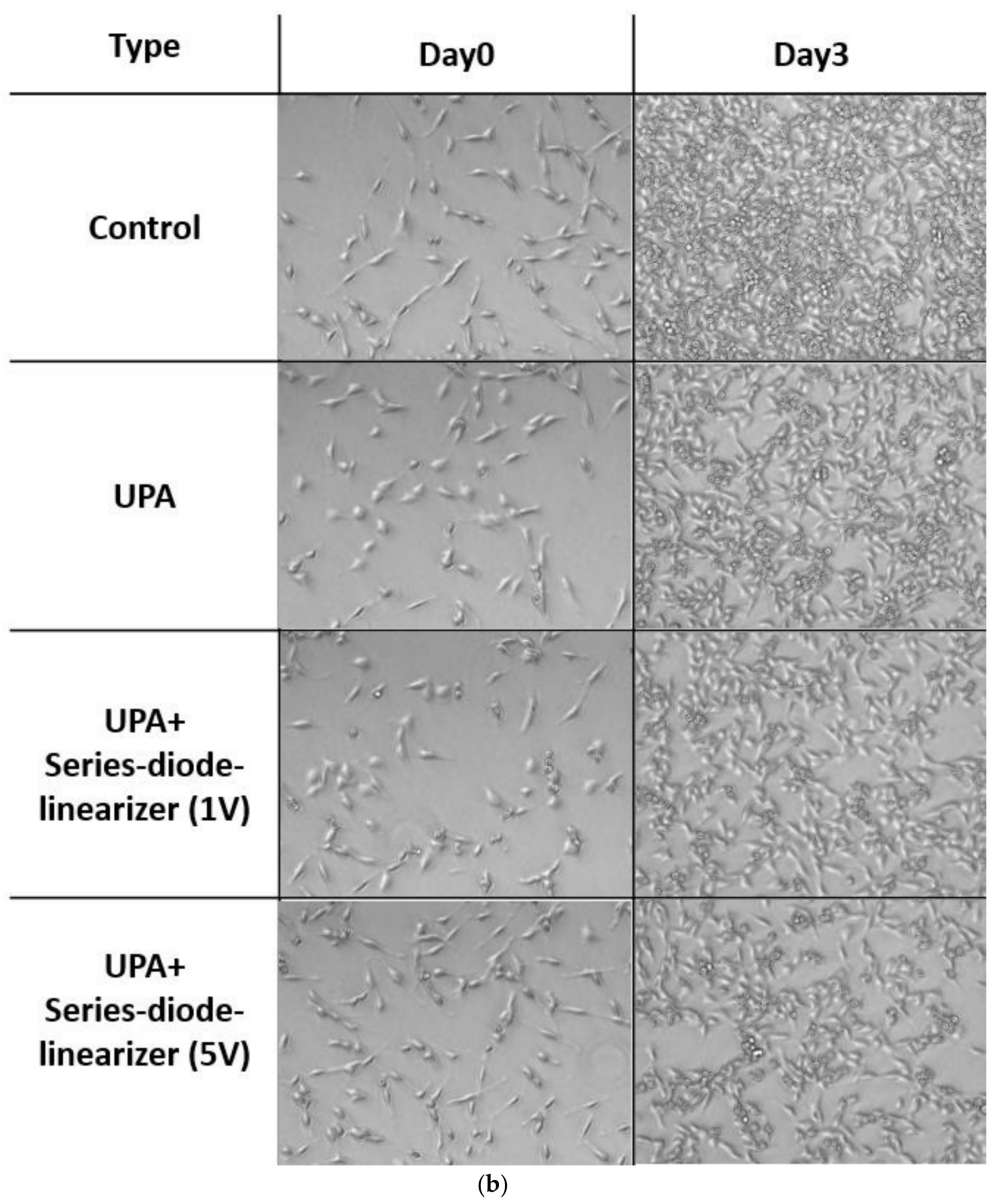
(**a**) HeLa cells experiment and (**b**) representative brightfield images from Day 0 to Day 3 acquired using a microscope.

The transmit intensity for different ultrasonic power amplifier topologies need to be quantified to be compared. The measured transmitted spatial average temporal average intensity (I_SATA_) of the ultrasonic transducers when using ultrasonic power amplifier only (UPA), ultrasonic power amplifier integrated with series-diode linearizer at 1 V DC (UPA + Series-diode linearizer (1 V)) and 5 V DC (UPA + Series-diode linearizer (1 V)) were 47.90, 82.15, and 128.11 mW/cm^2^, respectively. As shown in Figure 7a, 15 MHz pulses generated from the isolated ultrasonic power amplifier and the ultrasonic power amplifier integrated with the series-diode linearizer were sent through an expander. The performances of the ultrasonic power amplifier integrated with the series-diode linearizer at 1 V and 5 V DC voltages were compared with that of the isolated ultrasonic power amplifier to eventually compare the suppression effects of HeLa cell proliferation using the series-diode linearizer circuit. The output pulses were sent to a 15 MHz ultrasonic transducer with a focal distance of 0.5” (I2-1504-S-SU, Olympus-NDT, Waltham, MA, USA) to focus the ultrasound signals in the HeLa cells for 30 min daily. The stimulus-driven cell densities were measured using a microscope. Figure 7b shows the representative brightfield images acquired using inverted microscope under different experimental conditions.

**Figure 8:**
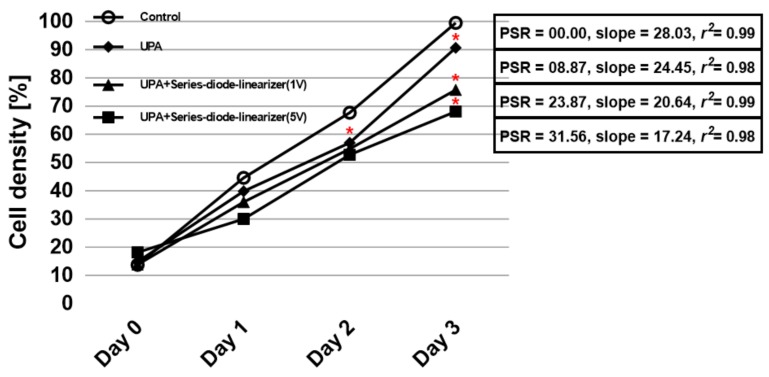
HeLa cells suppression experiments when exposed to the isolated ultrasonic power amplifier and the ultrasonic power amplifier integrated with series-diode linearizer from Day 0 to Day 3. SR refers to proliferation suppressing ratio. UPA, UPA + series-diode-linearizer (1 V), and UPA + series-diode-linearizer (5 V) represent ultrasonic induction when using the isolated ultrasonic power amplifier and the ultrasonic power amplifier with series-diode linearizer at 1 V DC bias voltage, and ultrasonic power amplifier with series-diode-linearizer at 5 V DC bias voltage, respectively. * *p* < 0.05.

Figure 8 shows the calculated cell densities from Figure 7b and the proliferation suppressing ratios between the control group with no ultrasonic stimulus and the experimental groups exposed to the ultrasound signals triggered by the isolated ultrasonic power amplifier and the ultrasonic power amplifier integrated with the series-diode linearizer at 1 V and 5 V DC bias voltages, respectively. According to the measured data, the experimental groups critically reduce the cell viability as compared to the control group. Specifically, in the experimental groups, HeLa cell densities, induced by the isolated ultrasonic power amplifier and the power amplifier integrated with the series-diode linearizer at 1 V and 5 V DC bias voltages, were decreased to 90.7 ± 1.2%, 75.8 ± 3.5%, and 68.1 ± 1.1%, respectively. In addition, their PSRs were 8.87%, 23.87%, and 31.56%, respectively. A simple linear regression was applied to the measured cell densities, and excellent linear correlations (*r*^2^ > 0.9) and significantly different slopes were achieved for experimental groups. Among the experimental groups, ultrasonic stimulus by the ultrasonic power amplifier integrated with the series-diode linearizer at 5 V DC bias voltage showed the most significant PSR (slope was 17.24, *r*^2^ = 0.98) as compared to the control group since the ultrasonic power amplifier with series-diode linearizer could boost the voltage gain than the ultrasonic power amplifier only for higher output power ranges. Table 1 summarizes the quantitative data of the cell densities from Day 0 to Day 3, PSRs on Day 3, and slope with *r*^2^ values using a simple linear regression.

## 4. Conclusions

The series-diode linearizer at certain DC bias voltages reduces the voltage variances at a high voltage output power, thus generating higher power into the ultrasonic transducers. To prove our proposed concept, we tested the pulse-echo responses when using the ultrasonic power amplifiers with and without the series-diode linearizer. The measured amplitude of the echo signals, when using the ultrasonic power amplifier integrated with the series-diode linearizer at 5 V DC voltage (0.128 V_p-p_), was higher than that when using the ultrasonic power amplifier only (0.0481 V_p-p_). A series-diode linearizer was proposed to improve the performances of the HeLa cell proliferation suppression. To verify the proposed idea for therapeutic applications, the isolated ultrasonic power amplifier and the ultrasonic power amplifier integrated with a series-diode linearizer were applied to HeLa cells and the therapeutic efficacies were observed. According to the results, the most significant changes were observed in the densities of HeLa cells when the ultrasonic power amplifier integrated with a series-diode linearizer at 5 V DC bias (68.1 ± 1.1%, PSR = 8.87%) was applied. Therefore, a series-diode linearizer can help an ultrasonic power amplifier to reduce cell density and increase PSR effectively by generating a higher output power with cleaner ultrasound waveforms.

## Figures and Tables

**Table 1:** Summary of quantitative data of cell densities with or without ultrasonic stimulus, PSRs, and slope with *r*^2^.

	Day 0	Day 1	Day 2	Day 3	PSR (vs. Control)	Slope	*r* ^2^
Control	13.8 ± 1.2%	44.6 ± 1%	67.7 ± 1.3%	99.5 ± 0.5%	0%	28.03	0.99
UPA	14.9 ± 1.9%	39.9 ± 3.1%	57.0 ± 1.4%	90.7 ± 1.2%	8.87%	24.45	0.98
UPA + Series-diode linearizer (1 V)	13.9 ± 2.4%	34.0 ± 3.1%	54.9 ± 2.0%	75.8 ± 3.5%	23.87%	20.64	0.99
UPA + Series-diode linearizer (5 V)	18.2 ± 1.8%	30 ± 0.9%	52.8 ± 3.2%	68.1 ± 1.1%	31.56%	17.24	0.98
